# Surgical Stabilisation of a Coxofemoral Luxation in a Northern Goshawk (*Accipter gentilis*) with Transarticular Pinning

**DOI:** 10.3390/vetsci10030205

**Published:** 2023-03-08

**Authors:** Marko Legler, Vanessa Guddorf, Michael Fehr

**Affiliations:** 1 Department of Small Mammal, Reptile and Avian Diseases, University of Veterinary Medicine Hannover, Foundation, Bünteweg 9, 30559 Hannover, Germany; 2 Seal Sanctuary and Rescue Centre, Dörper Weg 24, 26506 Norden-Norddeich, Germany

**Keywords:** luxation, bird of prey, hip joint

## Abstract

**Simple Summary:**

In hawks, luxation occurs infrequently compared with other orthopedic conditions. In this case, a northern goshawk was diagnosed with a luxation of the hip joint after an unsuccessful hunting flight. A surgical treatment of the luxation with an open reduction and transarticular pinning was performed. After healing and implant removal, the hawk could be successfully used for hunting again. The anatomical conditions of the hip joint and the advantages and disadvantages of a transarticular stabilization of this joint in the goshawk are discussed.

**Abstract:**

A 3-year-old male northern goshawk (*Accipiter gentilis*) used in falconry for hunting was diagnosed with a craniodorsal coxofemoral luxation of the left leg after an unsuccessful hunting flight. Closed reduction in the dislocation was unsuccessful and the hip joint reluxed again with slight abduction of the limb. An open surgical reduction with a transarticular stabilization using a normogradely inserted Kirschner wire was performed. The implant was removed surgically after five weeks. After about seven weeks, the owner saw no abnormalities in the loading of the limbs, and the goshawk was successfully used for hunting after nine months in the next hunting season.

## 1. Introduction

The northern goshawk is a popular bird of prey in falconry due to its uncompromising hunting style. The prey of this hawk consists of a wide range of birds and mammals. In captivity goshawks tend to be highly strung and nervous [1]. Luxations of the coxofemoral joint are described in different avian species, more frequently in birds weighing up to one kilogram. Among the more frequently affected avian taxa are also birds of prey. Compared to other orthopedic conditions luxations occur infrequently [2].

In hawks as non-cursorial species the hip joint is a complex diarthrodial joint with a prominent antitrochanter as an extension of the pelvis. The hip joint consists of two articular surfaces—the *Articulatio coxocapitalis* and the *Articulatio coxotrochanterica*. This arrangement of the hip joint as a gliding-hinge joint allows especially cranio-caudal movements and reduced lateral movements of the leg. The hip joint is stabilized by the intraarticular ligament of the femur head and three difficult to separate ligaments, the pubofemoral, iliofemoral, and ischiofemoral ligament [3,4,5].

In most cases, the etiology of a luxation of the hip joint is a trauma, for example due to improper handling. Predisposition for a luxation due to hip dysplasia especially in hand-reared birds has not been studied in detail [2]. 

Several procedures have been described to repair coxofemoral luxation in avian species [2,3]. Conservative management include spica splints, casts and slings [3,4,6,7,8,9]. Surgical stabilization can be achieved via polypropylene sutures [3,4,10], transarticular pinning [6] or toggle-pin technique [11]; excision arthroplasty of the femoral head has also been described [12,13,14].

The aim of this paper is to describe the successful management of a coxofemoral luxation in a northern goshawk. 

## 2. Case Presentation

### 2.1. History and Clinical Examination

**Figure 1:**
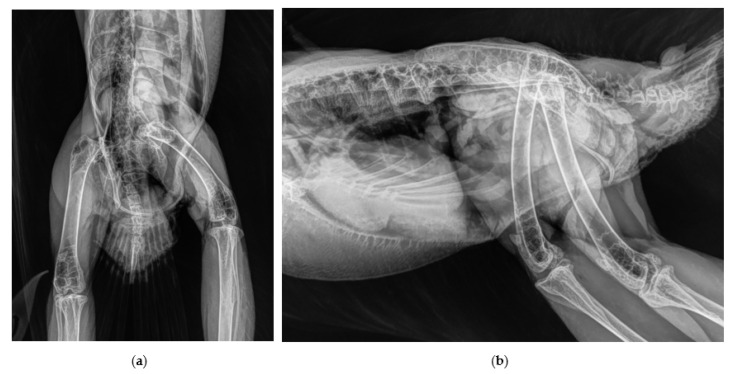
Cranio-dorsal luxation of the left coxofemoral joint, radiographs obtained in ventro-dorsal (**a**) and latero-lateral (**b**) position.

A captive breed, 3-year-old, male northern goshawk (*Accipiter gentilis gentilis*) weighing 750g used in falconry for crow hunting was presented with an injury at the department of small mammal, reptile and avian diseases of the University of Veterinary Medicine, Hannover. After an unsuccessful hunting flight, the goshawk was not able to put weight on the left hind limb and to use the leg properly when perching. The exact course of the accident could not be observed by the owner. On presentation, the bird was perching only on the right leg and the affected left hind limb was held in adduction and external rotation. Clinical examination revealed swelling of the trochanteric area, palpable crepitation during manipulation of the left hip joint and pain. The limb appeared shorter compared with the contralaterale. Orthogonal radiographic projection of the pelvis revealed a craniodorsal coxofemoral luxation (Figure 1).

**Figure 2:**
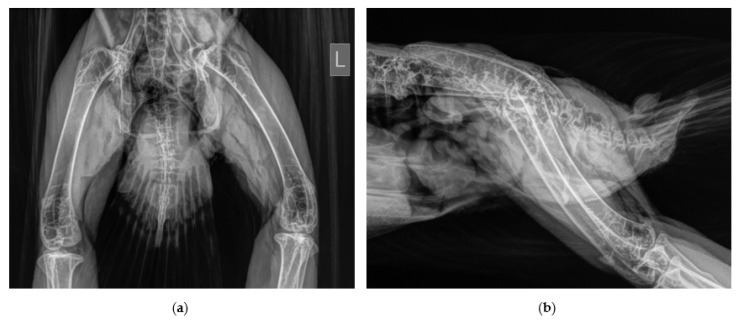
Subluxation of the left coxofemoral joint after closed reduction; radiographs obtained in ventro-dorsal (**a**) and latero-lateral (**b**) position.

Closed reduction in the coxofemoral joint in general anesthesia was performed and meloxicam (1 mg/kg; Metacam^®^ 2mg/mL, Boehringer Ingelheim Vetmedica GmbH, Ingelheim/Rhein, Germany) was administered intramuscularly. The femur was rotated externally to loosen the femur head and distal traction of the leg and after this the femur head was seat into the acetabulum by rotating the femur internally. Unfortunately, a subluxation of the affected joint could still be detected radiographically (Figure 2) and the hip reluxed even with slight abduction. Therefore, surgical stabilization was planned. 

### 2.2. Surgical Procedure and Post-Surgery Management

The goshawk was anesthetized with isoflurane (Isofluran CP^®^, CP Pharma GmbH, Burgdorf, Germany). The hawk was prepared for anesthesia by withdrawing food for 24 h and waiting until the pellet was thrown. As premedication, diazepam (0.5 mg/kg; Diazepam-ratiopharm^®^ 10 mg/2mL, ratiopharm GmbH, Ulm, Germany) and meloxicam (1 mg/kg; Metacam^®^ 2mg/mL, Boehringer Ingelheim Vetmedica GmbH, Ingelheim/Rhein, Germany) were administered intramuscularly. Anesthesia was induced with 5 volumen percent (vol.-%) isoflurane in 3 L/min oxygen with an anesthetic mask (Veterinary mask, Eickemeyer, Tuttlingen, Germany) and maintained after intubation with an uncuffed tracheal tube (2.5 Rüschelit^®^ SAFETY CLEAR 2,5 mm, Teleflex Medical GmbH, Fellbach, Germany) with 1.5–3.0 vol.-% isoflurane in 3 L/min oxygen and spontaneous breathing or manual ventilation if necessary in a semi-open anesthesia system with suction (Eickemeyer, Tuttlingen, Germany). The depth of anesthesia was calculated by toe pinch, wing twitch and corneal reflexes. 

The goshawk was positioned in lateral recumbency during surgery with the affected hip on top. All feathers of the hip joint region were removed, and the skin was disinfected with propanol (Cutasept^®^ F, BODE Chemie GmbH, Hamburg, Germany) and the side surrounding the surgery was covered with sterile gauze.

**Figure 3:**
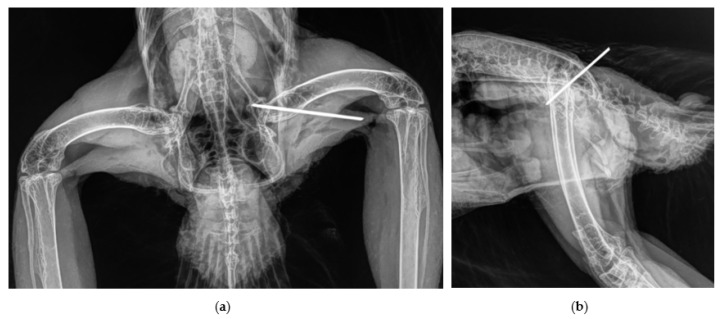
Intra-operative control of the position of the Kirschner wire in the repositionated coxofemoral joint; radiographs obtained in ventro-dorsal (**a**) and latero-lateral (**b**) position.

**Figure 4:**
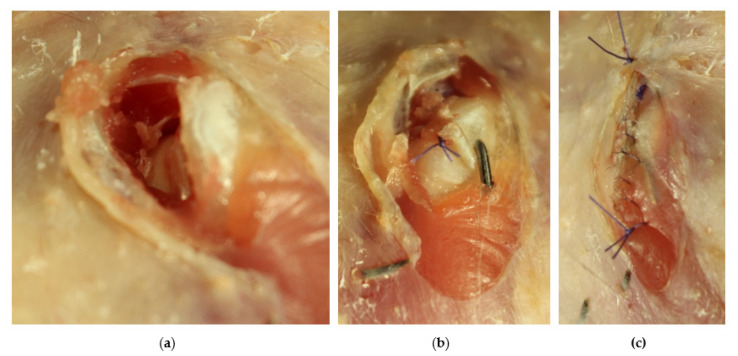
Craniolateral approach to the coxofemoral joint (**a**) with visible torn parts of the joint capsule and the femoral head and the wound closure with the suture of the *M. iliotrochantericus caudalis* and the bent Kirschner wire (**b**) and the suture of the *M. iliotibialis lateralis* and the started suture of the skin (**c**) (preparation of a Northern goshawk).

The luxated joint was approached using a craniolateral approach to the hip. After the skin incision, the muscles, *Musculus iliotibialis lateralis* and *Musculus iliotrochantericus caudalis*, were debrided longitudinally and the joint was visualized. The acetabulum and femoral head were inspected for further injuries and the luxation was reduced. Remnants of the round ligament and of the torn joint capsule and fibrin debris were removed from the acetabulum. After this, the head of the femur was held firmly in the acetabulum, with medially directed pressure and the joint held in slight abduction. A 1.0 mm Kirschner wire was inserted from the trochanter, through the femoral neck, to the acetabulum using a drill (CONMED/Linvatec Mpower2 PRO 6200M, CONMED Corporation, New York, USA). The pin length was estimated by measuring in the ventrodorsally radiograph. The proper position of the implant was confirmed radiographically and the Kirschner wire was bent at the femur and cut off and sunk under the musculature (Figure 3 and Figure 4). The remnants of joint capsule could be not closed and were left open for secondary healing. The musculature was sutured one by one continuously and the skin with single stitches. The implant restricted limb abduction as desired, and at the same time allowed good forward and backward movement.

Fluids (20 mL/kg, 1:1 Sterofundin^®^ ISO B. Braun Vet Care and Aminoplasmal^®^, B. BRAUN Melsungen AG, Melsungen, Germany) were administered subcutaneously. Non-steroidal anti-inflammatories (meloxicam, Metacam^®^, Boehringer Ingelheim Vetmedica GmbH, Ingelheim/Rhein, Germany; 1mg/kg BID for four days followed SID for six days), antibiotics (doxycycline, Ronaxan^®^, Boehringer Ingelheim Vetmedica GmbH, Ingelheim/Rhein, Germany; 50 mg/kg SID, 10 days) and antifungals (voriconazole, Voriconazol PUREN 50 mg, Puren Pharma GmbH and CoKG, 12.5 mg/kg bid) were orally prescribed. The goshawk had cage rest for eight days in in a shaded box that was closed on all sides with a towel as a soft and absorbent underlay. The hawk was then kept in a closed aviary 28 days until the control examination post-surgery. No special physiotherapy was performed during this time, the goshawk loaded the affected limb well and showed relief only at rest. The goshawk was transferred from a hunting weight (730 g) to a molting condition (850 g) in order to also control the goshawk’s temperament somewhat and to achieve better wound healing. The perches in the aviary were covered with astroturf to protect the bird from pododermatitis.

**Figure 5:**
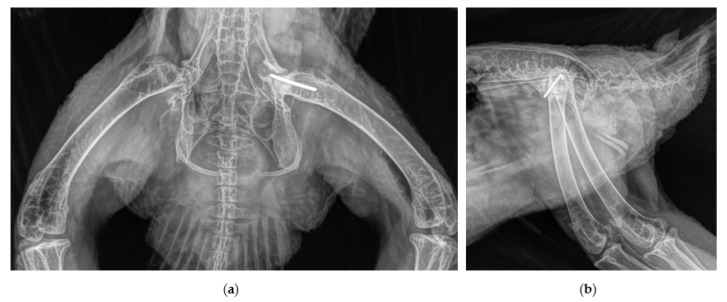
Five weeks post-surgery radiographically visible bone reaction behind the coxofemoral joint and no signs of reluxation; radiographs obtained in ventro-dorsal (**a**) and latero-lateral (**b**) position.

The radiographic examination under general anesthesia five weeks post-surgery revealed bone formation and reaction at the medial aspect of the acetabulum, a low-grade atrophy of the thigh musculature and no signs of luxation (Figure 5). A physiological movement of the leg was possible. The pin was removed in a second surgery with a small incision through the skin and the iliotibialis lateralis muscle. The wound was closed as previously described. Two weeks following implant removal, the owner did not see any difference comparing the load on both limbs, and after the molting session in which the hawk was kept in aviary the bird was used successfully for hunting again, in total, nine months after hip joint luxation. 

## 3. Discussion

**Figure 6:**
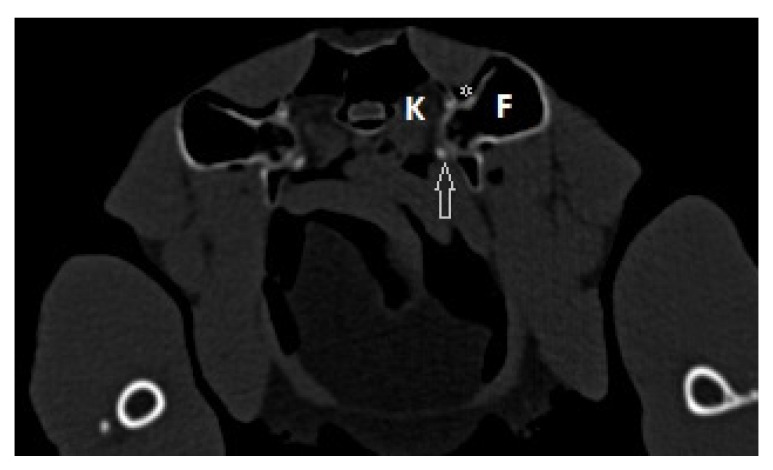
Computertomographic image of a northern goshawk at the level of the coxofemoral joints with the visible kidney (K), the femur (F) with the area of the fovea capitis and the insertion of the capitis femoris ligament (arrow) and the air sac around the coxofemoral joint (asterix).

The present case report describes the surgical treatment of a coxofemoral luxation in a Northern goshawk with the use of a transarticular pin after an unsuccessful conservative therapy. In our case the clinical signs with a forward adducted and slightly external rotated position of the leg with a reduced mobility are typical for a craniodorsal luxation of the coxofemoral joint, a very common luxation of hip joint in raptors [2,3,14]. The treatment of a luxation in general includes the reduction and stabilization of the joint to prevent reluxation. The stabilization of the coxofemoral joint by a transarticular pin has previously been described in small animal medicine, especially in cats, with a good long-term outcome without sacrificing the integrity of the coxofemoral joint [15]. This technique is also described in birds [2,6]. A possible complication in this surgical technique as well as in the toggle pin technique [10] in birds could be an injury of the kidney and the ischiadic nerve and artery with the transarticular pin [16,17]. All these anatomical structures are behind the acetabulum. Figure 6 demonstrate the arrangement of the anatomical structures around the coxofemoral joint. This figure shows that there is no relevant structure directly located behind the acetabulum in the area of the attachment of the intraarticular ligament in the northern goshawk. The transarticular pin should be passed through the joint in the course of the intraarticular ligament in order to minimize the risk of injuries and to maintain the greatest possible physiological mobility of the hip joint [15]. 

**Figure 7:**
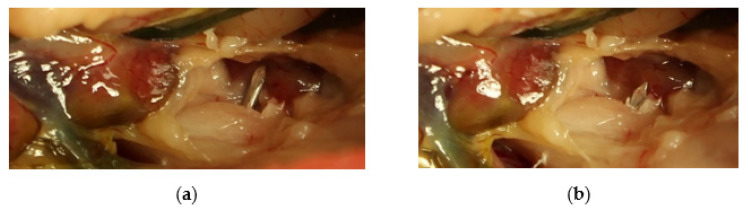
Movement of the transarticular pin tip of the coxofemoral joint depending on adduction (**a**) and abduction (**b**) of the leg during the preparation of a dead female northern goshawk. In this preparation the kidney was not injured by the Kirschner wire.

In addition, there is a movement of the pin tip during the movement of the leg. This can be seen in Figure 7, which shows the movement of the pin tip depending on the movement of the leg using a preparation of a Northern goshawk. In the control radiographs, in our case, a funnel-shaped bone formation could be seen behind the acetabulum, probably corresponding to the movement of the pin tip. The body probably tried to separate the tip of the pin as a foreign body. The movement also leads to strong forces applied to the pin in this region, which can lead to pin breakage.

However, in our case and in our preparations, there were no signs of a kidney injury in male and female northern goshawks. The anatomical conditions may be different in other individuals or in other subspecies. This should be considered as a risk factor. 

A coxofemoral luxation typically include the rupture of the articular capsule as well as the ligaments. The main advantage of an open surgical treatment is the complete removal of all parts of the joint capsule from the joint space and the resulting good fit of the femoral head in the joint socket. In particular, in the case of a hip luxation, the remaining parts of the joint capsule in particular can promote the development of arthrosis [2,16]. The lack of closure of the joint capsule in our case does not appear to have brought any disadvantages. Prompt treatment is important to keep cartilage damage and other injuries as low as possible. Arthrosis development is reported to start three days after luxation [16]. To ensure normal mobility of the joint, early mobilization and physiotherapy should be performed [2]. However, in our case, no special physiotherapy was carried out. The hawk used the affected leg well and we tried to keep the hawk in such a way that it did not overload the leg. The craniolateral approach to the hip joint was without complications in our case. The air sac around the joint makes it easier to see the joint space (Figure 4 and Figure 6). A caudolateral approach to the joint is reported which corresponding to the literature [17,18], and in our view carries an increased risk of injury to large blood vessels and nerves. 

However, further evaluations of clinical cases are necessary to validate this technique in raptors and find the most appropriate surgical method also for other avian species. 

## 4. Conclusions

The transarticular pinning technique appeared to be an effective treatment option to stabilize a luxated coxofemoral joint in northern goshawks. The craniolateral approach to the hip joint is a minimally invasive approach to the joint. However, the individual anatomical conditions should be taken into account in order to prevent injuries caused by the surgical technique. Surgical treatment should be considered, especially in cases where conservative treatment has not been successful.

## Data Availability

Not applicable.

## References

[1] McDermott M. (2004). Accipitrine Behavioral Problems: Diagnosis & Treatment.

[2] Azmanis P.N., Wernick M.B., Hatt J.-M. (2014). Avian Luxations: Occurrence, diagnosis and treatment. Vet. Q..

[3] Gonzales M.S. (2019). Avian articular orthopedics. Vet. Clin. N. Am. Exot. Anim. Pract..

[4] Martin H.D., Kabler R., Sealing L. (1994). The avian coxofemoral joint: A review of regional anatomy and report of an open reduction technique for repair of a coxofemoral luxation. J. Assoc. Avian Vet..

[5] Baumel J.J., Raikow R.J., Baumel J.J. (1993). Arthrologia. Handbook of Avian Anatomy: Nomina Anatomica Avium.

[6] Roush J.C., Kirk R.W. (1980). Avian orthopedics. Current Veterinary Therapy.

[7] Altman R.B., Petrak M.L. (1982). Disorders of the skeletal system. Diseases in Cage and Aviary Birds.

[8] Mac Coy D.M. (1989). Excision arthroplasty for management of coxofemoral luxation in pet birds. J. Am. Vet. Med. Assoc..

[9] Stauber E., Mulholland J.A., Levine E.W., Suzuki Y., Hall J. (2008). Succesfull rehabilitation of a severely injured peregrine falcon. J. Avian Med. Surg..

[10] Risi E., Ordonneau D., Gauthier O. Two cases femoral head luxations in cormorants (*Phalacrocorax carbo*) treated with modified Meij-Hazewinkel-Nap technique. Proceedings of the European Association of Avian Veterinarians.

[11] Yazici M.F. (2020). Surgical correction of a coxofenoral luxation in a European Honey buzzard (*Pernis apivorus*) by incorporating a toggle-pin technique: A case report. J. Exot. Pet. Med..

[12] Ackermann J., Porter S. (1995). Femoral head osteotomy in a red-shouldered hawk (*Buteo lineatus*). J. Avian Med. Surg..

[13] Campbell T.W. Excision arthroplasty in a Toco tucan (*Ramphastos toco toco*) for the correction of an osteoarthritis of the right coxofemoral joint. Proceedings of the First Conference on Zoological and Avian Medicine of the Association of Avian Veterinarians and American Association of Zoo Veterinarians.

[14] Moisuk-Burgdorf A., Whittington J.K., Bennett R.A., McFaden M., Mitchell M., Obrien R. (2011). Successful management of simple fractures of the femoral neck withfemoral head and neck excision arthroplasty in two free-living avian species. J. Avian Med. Surg..

[15] Sissener T.R., Whitelock R.G., Langley-Hobbs S.J. (2009). Long-term results of transarticular pinning for surgical stabilization of coxofemoral luxation in 20 cats. J. Small Anim. Pract..

[16] Bennett R.A., Altman B.R., Altman B.R., Clubb L.S., Dorrestein G.M., Quesenberry K. (1997). Orthopedic surgery. Avian Medicine.

[17] Hatt J.-M., Chitty J., Lierz M. (2008). Hard tissue surgery. BSAVA Manual of Raptors, Pigeons and Passerine Birds.

[18] Harcourt-Brown N.H. (2002). Orthopedic conditions that affect the avian pelvic limb. Vet. Clin. N. Am. Exot. Anim. Pract..

